# Annexin A1 Mimetic Peptide and Piperlongumine: Anti-Inflammatory Profiles in Endotoxin-Induced Uveitis

**DOI:** 10.3390/cells10113170

**Published:** 2021-11-15

**Authors:** Ana Paula Girol, Caroline de Freitas Zanon, Ícaro Putinhon Caruso, Sara de Souza Costa, Helena Ribeiro Souza, Marinônio Lopes Cornélio, Sonia Maria Oliani

**Affiliations:** 1 Department of Physical and Morphological Sciences, University Center Padre Albino (UNIFIPA), Catanduva 15809-144, SP, Brazil; anapaula.girol@unifipa.com.br (A.P.G.); sarah_sc_0705@hotmail.com (S.d.S.C.); helena.souza@unifipa.com.br (H.R.S.); 2 Department of Biology, Institute of Biosciences, Humanities and Exact Sciences (Ibilce), São Paulo State University, (UNESP), São José do Rio Preto 15054-000, SP, Brazil; carolfzanon@yahoo.com.br; 3 Post Graduate Program in Structural and Functional Biology, Escola Paulista de Medicina (UNIFESP-EPM), Federal University of São Paulo, São Paulo 04023-062, SP, Brazil; 4 Department of Phisics, Institute of Biosciences, Humanities and Exact Sciences (Ibilce), São Paulo State University, (UNESP), São José do Rio Preto 15054-000, SP, Brazil; icaro.caruso@unesp.br (Í.P.C.); mariol.cornelio@gmail.com (M.L.C.); 5 Advanced Research Center in Medicine (CEPAM), União das Faculdades dos Grandes Lagos (Unilago), São José do Rio Preto 15030-070, SP, Brazil

**Keywords:** eye inflammation, lipopolysaccharide, natural bioactive extracts, Ac2-26, FPR receptor, inflammatory mediators

## Abstract

Uveitis is one of the main causes of blindness worldwide, and therapeutic alternatives are worthy of study. We investigated the effects of piperlongumine (PL) and/or annexin A1 (AnxA1) mimetic peptide Ac2-26 on endotoxin-induced uveitis (EIU). Rats were inoculated with lipopolysaccharide (LPS) and intraperitoneally treated with Ac2-26 (200 µg), PL (200 and 400 µg), or Ac2-26 + PL after 15 min. Then, 24 h after LPS inoculation, leukocytes in aqueous humor, mononuclear cells, AnxA1, formyl peptide receptor (fpr)1, fpr2, and cyclooxygenase (COX)-2 were evaluated in the ocular tissues, along with inflammatory mediators in the blood and macerated supernatant. Decreased leukocyte influx, levels of inflammatory mediators, and COX-2 expression confirmed the anti-inflammatory actions of the peptide and pointed to the protective effects of PL at higher dosage. However, when PL and Ac2-26 were administered in combination, the inflammatory potential was lost. AnxA1 expression was elevated among groups treated with PL or Ac2-26 + PL but reduced after treatment with Ac2-26. Fpr2 expression was increased only in untreated EIU and Ac2-26 groups. The interaction between Ac2-26 and PL negatively affected the anti-inflammatory action of Ac2-26 or PL. We emphasize that the anti-inflammatory effects of PL can be used as a therapeutic strategy to protect against uveitis.

## 1. Introduction

Uveitis is an intraocular inflammation of different etiologies [1,2,3,4,5] characterized by leukocyte accumulation in ocular tissues and cytokine release. It is a painful condition and is associated with redness, photophobia, impaired vision, and blindness [6,7,8,9,10]

Pharmacological treatment for uveitis includes corticosteroids, chemotherapeutic agents, and tumor necrosis factor (TNF)-α inhibitors [3,7,8,11], but the use of these drugs is limited by their serious side effects, such as increased ocular pressure or cytotoxicity [9,12]. However, recent advances in the mechanisms of inflammation and the discovery of several endogenous anti-inflammatory mediators have provided new therapeutic possibilities for uveitis treatment [5,7,10,13,14,15].

In particular, the endogenous protein annexin A1 (AnxA1) may represent an alternative therapy for uveitis [16,17,18,19,20]. AnxA1 is an anti-inflammatory 37 kDa protein, which exhibits calcium and membrane phospholipid binding sites and is involved in the inhibition of glucocorticoid-induced eicosanoids and phospholipase A2 synthesis [21,22,23,24]. Structurally, AnxA1 comprises a specific small N-terminal region and a central domain formed by four to eight replicates of a highly conserved 70 to 80 amino acids sequence [25,26,27]. The N-terminal domain contains sites for post-translational processes, such as phosphorylation, glycosylation, and proteolysis [17,24,28,29].

Over the years, our research group has investigated the effect of AnxA1 on different ocular inflammatory conditions [17,19,30,31,32,33,34,35]. Positive modulation of endogenous AnxA1 in inflammatory cells in the eyes of mice and retinal pigment epithelial cells (ARPE-19) infected with *Toxoplasma gondii* suggests the protein can be used as a therapeutic target in ocular toxoplasmosis [30]. AnxA1 is involved in the signaling cascades of inflammatory processes, leading to decreased cell proliferation and increased migration by modulation of connective tissue growth factor (CTGF) and lecithin retinol acyltransferase (LRAT) gene expression in ARPE-19 cells [19]. In an experimental allergic conjunctivitis model using wild and AnxA1-null Balb/c mice, administration of the AnxA1-N-terminal region mimetic peptide (Ac2-26) was effective in reducing interleukin (IL)-2, IL-4, IL-10, IL-13, eotaxin, and regulated upon activation normal T cell expressed and presumably secreted (RANTES) [32]. In addition, the potential involvement of the formyl peptide receptor (fpr) family in the protective effect of Ac2-26 was investigated in the same allergic conjunctivitis model [35]. In a *Pseudomonas aeruginosa* keratitis model, the overexpression of AnxA1 and fpr2 occurred in the corneas of Balb/c mice and especially C57BL/6 mice, which is more susceptible to pathogens and infectious antigens [34]. Concerning the uveitis, the expression of AnxA1 in leukocytes and aqueous humor (AqH) was observed in endotoxin-induced uveitis (EIU) in rats [16], with this protein noted as one of the essential mediators in the inflammatory homeostasis process. Moreover, the mechanism of action and potential use of AnxA1 and Ac2-26 were demonstrated in EIU in rodents and lipopolysaccharide (LPS)-activated ARPE-19 cells [17]. The results of this investigation showed that following specific serine phosphorylation, AnxA1 can be translocated to the cell surface, where it interacts with fpr2 and inhibits the release of inflammatory mediators independent of the nuclear factor (NF)-kB signaling pathway and in a post-translational manner.

In recent years, another potent anti-inflammatory mediator, piperlongumine (PL) (5,6-dihydro-1-[(2E)-1-oxo-3-(3,4,5-trimethoxyphenyl)-2-propenyl]-2(1H)pyridinone), a biologically active component of *Piper* species (Piperaceae), has attracted the attention of our research group for its possible interaction with AnxA1 [36]. In particular, PL is the main alkaloid of long pepper (*Piper longum* L.), and its pharmacological actions include cytotoxic, antitumor, antiangiogenic, antiplatelet, antibacterial, antidiabetic, antianxiolytic, antiatherosclerotic, and antifungal effects [36,37,38,39,40,41,42,43]. PL induces apoptosis by interfering with redox and reactive oxygen species (ROS) homeostatic regulators, such as glutathione S-transferase pi 1 (GSTP1) and carbonyl reductase (CBR1) [44]. Moreover, on nonsmall cell lung cancer (NSCLC) in vivo and in vitro, *PL* suppressed lung cancer cell growth in a dose-dependent manner via inhibition of the NF-κB signaling pathway [45].

Regarding inflammation, LPS insult on PL protected the vascular barrier integrity by inhibiting hyperpermeability, expression of cellular adhesion molecules (CAMs), and adhesion and migration of leukocytes, thus endorsing its usefulness as a therapy for vascular inflammatory diseases [46]. Moreover, PL and derivatives reduced the amount of nitric oxide (NO) in LPS-stimulated RAW264.7 macrophages [47]. The protective effect of *P. longum* alkaloid extract containing piperine and PL on dopaminergic neurons against inflammatory reaction was observed in LPS-induced damage. The active extract attenuated the depletion of dopamine in the striatum, facilitated the survival of damaged neurons by inhibiting microglial activation, suppressed the release of neurotoxic factors, and improved LPS-induced behavioral dysfunctions [48]. For neuroinflammation caused by LPS in a model of amyloidogenesis, PL exhibited anti-inflammatory and antiamyloidogenic effects by inhibiting NF-κB [49].

Our investigations indicated that PL attenuated systemic and pulmonary inflammatory changes, partially by modulating the expression of the endogenous AnxA1, in lung inflammation induced by cigarette smoke [42]. The potential of PL as a therapeutic immunomodulator for cancer prevention and progression was reinforced by analyzing PL administration in human cancer cells from an epidermoid carcinoma of the larynx (Hep-2) and umbilical vein endothelial cells (HUVEC), in which PL modulated the expression of genes involved in inflammatory processes [36]. Although there are several publications related to PL, few studies have focused on its anti-inflammatory role, and the actions of PL in ocular inflammation are not known. Building upon these observations, we decided to investigate the role of PL as an alternative therapy for EIU.

Ac2-26 and PL interaction has been explored by our group through different molecular or computational screening techniques, such as phage display and molecular docking [36]. In silico analyses showed that, among other PL molecules, there was a terpene that appeared to interact with lysine 9 from AnxA1 in the region corresponding to the N-terminal peptide Ac2-26 [36]. However, the physiological reason for this interaction, whether positive or negative in vivo, with regard to the anti-inflammatory effects of AnxA1 is not yet understood, which opens up a new and stimulating field for research.

Given the above, we tested PL in EIU either alone or in coadministration with the peptide Ac2-26, followed by analyses of the leukocyte influx and inflammatory mediators, to verify the following hypotheses: (1) there is anti-inflammatory potential of PL in EIU; (2) Ac2-26 + PL coadministration may interfere with the anti-inflammatory response profile of Ac2-26, favoring or attenuating the effects of its administration on experimental uveitis.

## 2. Materials and Methods

### 2.1. Experimental Model of Uveitis and Treatment Protocols

Male Wistar rats, 6 to 8 weeks of age (200 g), were distributed in 7 groups (*n* = 10/group). The animals were kept in cages in a temperature-controlled environment (22 to 25 °C) and received water and food ad libitum. The experimental procedures were conducted according to the guidelines for biomedical research stated by the Brazilian Societies of Experimental Biology and also approved by the Ethic Committee on Animal Use of University Center Padre Albino (Certificate (No. 11/14). The experiments were designed to minimize the number of animals used and their suffering during the execution of the protocols. All animals were evaluated daily by the institution’s veterinarian.

For the development of EIU, rats were anesthetized with isoflurane (1%) and inoculated subcutaneously in the right footpad with 200 μg (1 mg/kg) of lipopolysaccharide (LPS type *Escherichia coli* serotype 0127: B8, Sigma Chemical Co. Poole, Dorset, UK) diluted in 100 μL of phosphate-buffered saline (PBS) [16,17].

**Figure 1:**
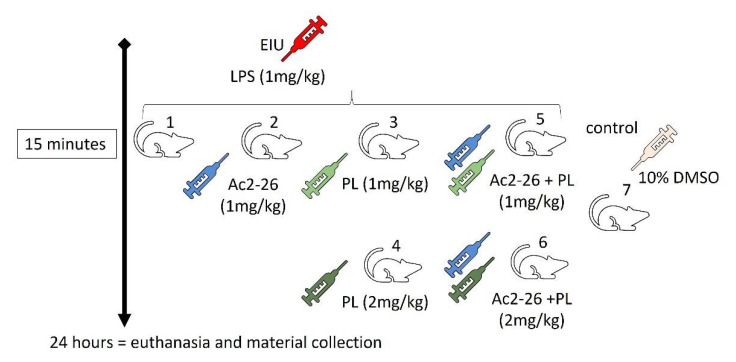
Schematic representation of experimental groups. Induced by (**1**) LPS, (**2**) LPS and treated with Ac2-26 200 μg diluted in PBS, (**3**) LPS and treated with PL 200 μg diluted in 10% DMSO, (**4**) LPS and treated with PL 400 μg diluted in 10% DMSO, (**5**) LPS and treated with Ac2-26 200 μg diluted in PBS and PL 200 μg diluted in 10% DMSO, (**6**) LPS and treated with Ac2-26 200 μg diluted in PBS and PL 400 μg diluted in 10% DMSO, and (**7**) uninduced, administered with 10% DMSO (*n* = 5/group).

The anti-inflammatory effects of Ac2-26 peptide (Ac-AMVSEFLKQAWFIENEEQEYVQTVK, Thermo Fisher Scientific, Grand Island, NY, USA) and PL (C_17_H_19_NO_5_, CAS number: 20069-09-4, Sigma-Aldrich/Merck, Darmstadt, Hesse, Germany) administered singly or in combination were tested by intraperitoneal (ip) injection 15 min after LPS induction in five EIU groups (*n* = 10/group) (Figure 1) [17]. The dosage of Ac2-26 at 200 μg (1 mg/kg or 539 μM) diluted in 100 μL of PBS was based on previous studies [17]. For the selection of the PL dosage and for the purpose of comparison with the peptide, we chose two dosages. The lowest dosage of 200 μg (1 mg/kg or 1.57 mM) was equal to the one used for the peptide, while the highest dosage was based on another investigation by our group, which tested the therapeutic efficacy of PL by ip administration at a dosage of 400 μg (2 mg/kg or 3.15 mM) diluted in 100 μL of 10% dimethyl sulfoxide (DMSO, Gold Lab; Ribeirão Preto, São Paulo, Brazil) [42]

Rats that received an intraperitoneal injection of 10% DMSO were used as the control group. The animals were anesthetized with isoflurane (1%) before each experimental treatment and euthanized 24 h after LPS inoculation by excessive dose of the anesthetic.

### 2.2. Histopathological and Quantitative Analyses

AqH was collected by puncturing the anterior chamber of the left eyes, and 10 μL samples were used and stained in Turk’s solution (90 μL). Blood was collected by cardiac puncture, and 10 μL samples were diluted in 190 μL of Turk’s solution. Neutrophils and monocytes were quantified in the Neubauer chamber. Values for quantification of AqH and blood leukocytes were expressed as mean ± standard error (SEM) of the average number of cells × 10^5^/mL in the AqH samples and the number of cells × 10^3^/mL in the blood samples [31].

After AqH collection, the left eyes were fixed in 4% formaldehyde, dehydrated in increasing order of alcohol content, and placed in paraffin for histopathological analyses and immunohistochemistry. These analyses were performed in the Leica DM500 microscope (Leica, Wetzlar, Hessen, Germany).

### 2.3. Immunohistochemical and Densitometric Studies

Detection of the AnxA1 protein, fpr1 and fpr2 receptors, cyclooxygenase (COX)-2 enzyme, and phagocytic mononuclear cells (macrophages and monocytes) were performed in 5 μm sections of the paraffin-embedded material. After an antigen retrieval step using citrate buffer (pH 6), the endogenous peroxide activity was blocked, and the sections were incubated overnight at 4 °C with the primary rabbit polyclonal antibodies anti-AnxA1 (1:2000) (Invitrogen, Camarillo, CA, USA), anti-fpr1 and anti-fpr2 (1:1000) (Bioss Inc., Wo-burn, MA, USA), anti-COX-2 (1:1000) (Bioss Inc, Wo-burn, MA, USA) and with monoclonal anti-ED-1 (monocytes and macrophages) (1:1000) (Millipore, Temecula, CA, USA) diluted in 1% BSA. Subsequently, the slides were incubated with biotinylated secondary antibody (Histostain kit, Invitrogen, Carlsbad, CA, USA). Positive staining was detected using a peroxidase-conjugated streptavidin complex, and color was developed using diaminobenzidine substrate (DAB Kit, Invitrogen, Carlsbad, CA, USA). The sections were counterstained with hematoxylin.

ED-1 positive cells were quantified in the anterior segment of the eyes of different groups with the aid of the Leica Image Analysis software, and the values obtained were expressed as number of cells per mm^2^. For the protein densitometric analyses, 3 different slides from each animal were used, and 15 points were analyzed in 3 regions of the cornea, iris, and ciliary processes for an average related to the intensity of immunoreactivity. The values were obtained as arbitrary units [17].

### 2.4. Inflammatory Mediator Levels

The intact right eyes of all the studied groups were macerated in liquid nitrogen and placed in eppendorfs, which were added with 500 μL of protease (Protease Inhibitor Cocktail Set I, Cat. No. 53391, Millipore Corporation, CA, USA) and phosphatase (PhosphoSafe, Cat. No. 7,126-3-3, Novagen, Millipore Corporation, Billerica, CA, USA) inhibitor solution prepared according to the manufacturer’s instructions. The material was incubated for 20 min at 4 °C under constant stirring and centrifuged at 14,000 RPM for 10 min at 4 °C. The supernatants were then collected and immediately frozen at −80 °C. The protein concentration in the supernatant was measured using a Bradford assay (Bio-Rad, Hemel Hempstead, UK).

IL-1β, IL-6, IL-10, monocyte chemoattractant protein (MCP)-1, and TNF-α inflammatory mediators were quantified in the eye macerate supernatant and in blood plasma using the rat cytokine MILLIPLEX MAP Kit (RECYTMAG-65K; Millipore Corporation, Billerica, CA, USA) according to the manufacturer’s instructions and analyzed on the LUMINEX xMAP MAGPIX (Millipore Corporation, Billerica, CA, USA) equipment. The concentration of analytes was determined by MAGPIX xPONENT software (Millipore Corporation, Billerica, CA, USA). Results were expressed as mean ± SEM of cytokine concentrations (pg/mL).

### 2.5. Statistical Analyses

The results were first submitted to descriptive analysis and normality determination. As the samples presented normal distribution, the analysis of variance (ANOVA) was used, followed by the Bonferroni test. All values were expressed as mean ± SEM and *p* values less than 0.05 were considered statistically significant.

## 3. Results

### 3.1. Singly Administered, the Treatments Inhibited the Influx of Leukocytes, Indicating Protective Effects of PL, Especially at 400 μg Dosage, and Confirming the Anti-Inflammatory Action of Ac2-26, but These Effects Were Lost with Coadministration

**Figure 2:**
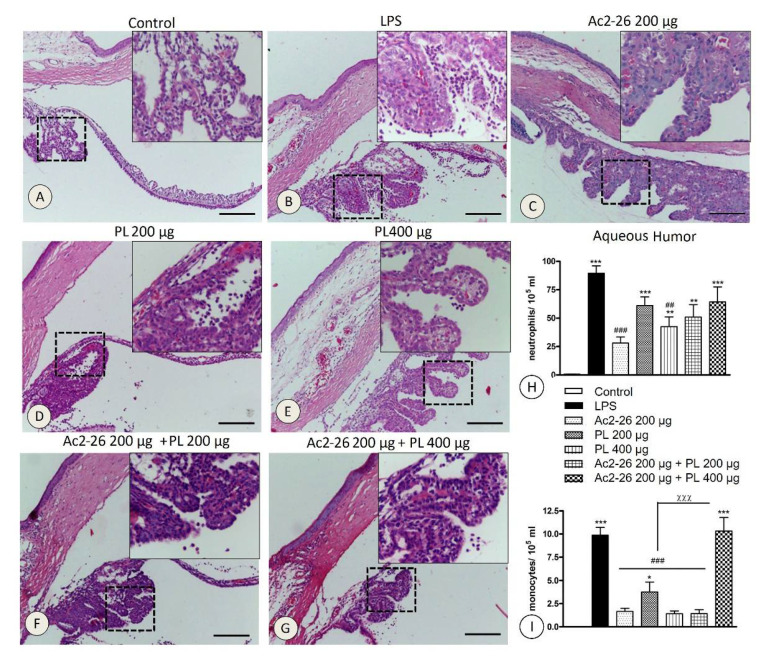
Histopathological analyses of ocular tissues in EIU. Absence of leukocytes in control tissues (**A**). Influx of neutrophils after 24 h LPS (**B**) and treated with Ac2-26 + PL 400 μg (**G**). Significant decrease in cellular extravasation after systemic treatments with Ac2-26 (**C**) and PL 400 μg (**E**) and moderate inflammatory influx reduction after treatments with PL 200 μg (**D**) and Ac2-26 + PL 200 μg (**F**). The details show enlargements of dashed areas. Sections: 5 μm, stain: HE, bars: 100 μm. Quantitative analyses of neutrophils (**H**) and monocytes (**I**) in the aqueous humor. The data show mean ± SEM of neutrophils and monocytes × 10^5^ mL in the eyes of control, untreated (LPS), and treated (Ac2-26 200 µg, PL 200 µg, PL 400 µg, Ac2-26 + PL 200 µg, and Ac2-26+ PL 400 µg) rats (*n* = 10 animals/group). *** *p* < 0.001, ** *p* < 0.01, and * *p <* 0.05 versus control; ### *p* < 0.001 and ## *p* < 0.01 versus LPS; and χχχ *p* < 0.001 versus Ac2-26 200 µg, PL 200 µg, PL 400 µg, and Ac2-26 200 µg + PL 200 µg.

Transmigrated leukocytes were absent in the control eyes (Figure 2A), but a high influx of these cells, mainly neutrophils, occurred 24 h after LPS inoculation without treatment (Figure 2B). The anterior eye segment was the most affected, and the inflammatory cells were observed in AqH, anterior and posterior chambers, and also in iris, ciliary body, and ciliary process stroma (Figure 2B). Except for Ac2-26 + PL 400 μg group (Figure 2G), fewer transmigrated leukocytes were presented after treatments (Figure 2C–F), especially in Ac2-26 (Figure 2C) and PL 400 μg (Figure 2E) groups.

**Figure 3:**
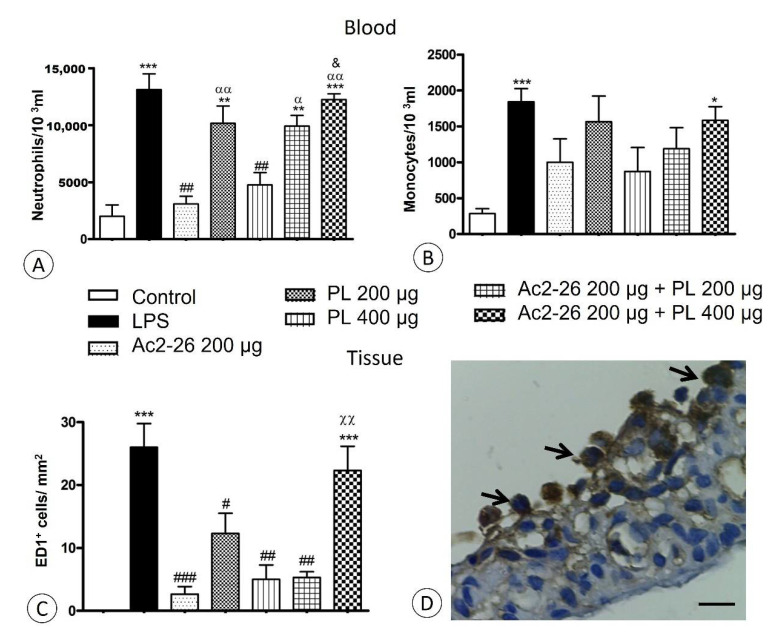
Quantitative analyses of neutrophils (**A**) and monocytes (**B**) in blood. Data show mean ± SEM of neutrophils and monocytes × 10^3^ mL in the blood of control, untreated (LPS), and treated (Ac2-26 200 µg, PL 200 µg, PL 400 µg, Ac2-26 + PL 200 µg, and Ac2-26 + PL 400 µg) rats (*n* = 10 animals/group). *** *p <* 0.001, ** *p* < 0.01, and * *p <* 0.05 versus control; ## *p <* 0.01 versus LPS; αα *p* < 0.01 and α *p* < 0.05 versus Ac2-26 200 µg; and & *p* < 0.05 versus PL 400 µg. Quantification of phagocytic mononuclear cells in the anterior segment of the eye (**C**). Data show mean ± SEM of ED-1 positive cells per mm^2^ in the eyes of control, untreated (LPS) and treated (Ac2-26 200 µg, PL 200 µg, PL 400 µg, Ac2-26 + PL 200 µg and Ac2-26 + PL 400 µg) rats. (*n* = 10 animals/group). *** *p <* 0.001 versus control; # *p <* 0.05, ## *p* < 0.01, and ### *p* < 0.001 versus LPS; and χχ *p <* 0.01 versus Ac2-26 200 µg, PL 400 µg, and Ac2-26 200 µg + PL 200 µg. ED-1 positive cells (arrows) on iris induced to uveitis and untreated (**D**). Sections: 5 μm, counterstain: hematoxylin, bars: 10 μm.

Decreased neutrophil transmigration into AqH was verified after Ac2-26 (*p* < 0.001) and PL 400 μg (*p* < 0.01) administrations compared to the untreated LPS group (Figure 2H). The influx of monocytes into AqH was also reduced by treatments (*p* < 0.001), except in the Ac2-26 + PL 400 μg group (Figure 2I). Similarly, in blood quantifications, higher numbers of neutrophils and monocytes were observed, especially in the LPS and Ac2-26 + PL 400 μg groups, with a marked reduction after Ac2-26 and PL 400 μg treatments (Figure 3A,B).

Phagocytic mononuclear cells were studied following immunohistochemical reaction with anti-ED-1 antibody in the ciliary body and iris (Figure 3D). Quantification showed a large number of ED-1 positive cells in the LPS and Ac2-26 + PL 400 μg (Figure 3C) groups (*p* < 0.001) compared to the control but significant decrease in the other groups.

Analyses related to influx of neutrophils and monocytes in AqH and blood and phagocytic mononuclear cells in the anterior eye segment showed anti-inflammatory action of Ac2-26 and dose-dependent effects of PL, which was more efficient at 400 μg dosage. In contrast, Ac2-26 + PL coadministration abrogated the peptide inhibitory action on neutrophil and monocyte extravasation, especially in the Ac2-26 + PL 400 μg group.

### 3.2. Ac2-26 and PL Singly Administered Reduced the Release of Proinflammatory Mediators in EIU, but These Effects Were Abrogated with Coadministration, Especially Ac2-26 + PL 400 μg

**Figure 4:**
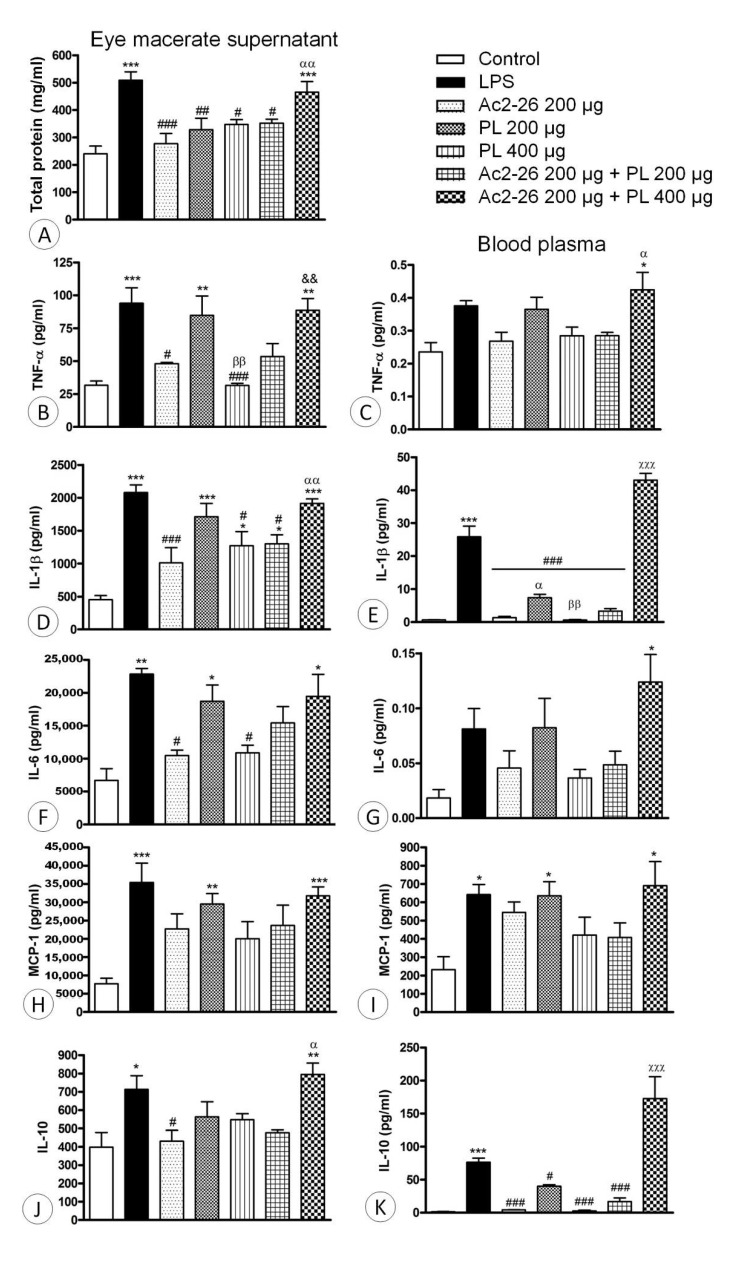
Effects of peptide Ac2-26 and PL, administered singly or in combination, on EIU. Levels of total proteins in supernatants after maceration of ocular tissues (**A**) and dosages of anti-inflammatory mediators TNF-α (**B**,**C**), IL-1β (**D**,**E**), IL-6 (**F**,**G**), and MCP-1 (**H**,**I**) and proinflammatory cytokine IL-10 (**J**,**K**) in the macerated eye supernatant and blood plasma. Data expressed as mean ± SEM of mg of proteins/mL and pg of cytokines/mL of control, untreated (LPS), and treated (Ac2-26 200 µg, PL 200 µg, PL 400 µg, Ac2-26 + PL 200 µg, and Ac2-26 + PL 400 µg) rats (*n* = 10 animals/group). *** *p* < 0.001, ** *p* < 0.01, and * *p* < 0.05 versus control; # *p* < 0.05, ## *p* < 0.01, and ### *p* < 0.001 versus LPS; αα *p* < 0.01 and α *p* < 0.05 versus Ac2-26 200 µg; ββ *p* < 0.01 versus PL 200 µg; && *p <* 0.01 versus PL 400 µg; and χχχ *p* < 0.001 versus all other groups.

The supernatants of macerated ocular tissues of LPS animals and those treated with Ac2-26 + PL 400 μg showed significant increase in total protein levels (*p <* 0.001) compared to the control. In the other treated groups, the protein concentration was reduced in relation to the LPS group (Figure 4A).

The proinflammatory mediators IL-1β, IL-6, TNF-α, and MCP-1 and the anti-inflammatory cytokine IL-10, which are all multifunctional molecules that play important roles in host defense in acute phase inflammatory reactions, were analyzed in supernatants of the ocular tissues after maceration and in the blood plasma. The results indicated low levels of the proinflammatory cytokines in the control eyes and, as expected, significant increased levels in the untreated LPS group, both in the macerated supernatant (Figure 4B,D,F,H) and blood plasma (Figure 4C,E,G,I).

Treatments with PL at 200 μg dosage singly administered or in combination with Ac2-26 peptide were able to reduce the levels of IL-1β only in the blood plasma (Figure 4E), while the combined administration of the AnxA1 mimetic peptide and PL at 400 μg dosage did not reduce the proinflammatory cytokine levels (Figure 4B–K). In contrast, when the peptide Ac2-26 or PL at 400 μg dosage were singly administered, IL-1β, IL-6, and TNF-α levels were reduced compared to the LPS group (Figure 4B–K), indicating resolution of the inflammatory process.

Regarding IL-10, increased levels were verified in the LPS and Ac2-26 + PL 400 μg groups in blood plasma and macerated supernatant (Figure 4J,K). Treatments with Ac2-26 peptide and PL (200 and 400 μg) reduced the cytokine concentration in blood plasma and eye supernatant, while Ac2-26 + PL 200 μg administration decreased IL-10 in blood plasma.

### 3.3. COX-2 Expression Is Not Inhibited after Treatments with PL 200 μg and Ac2-26 + PL 400 μg

**Figure 5:**
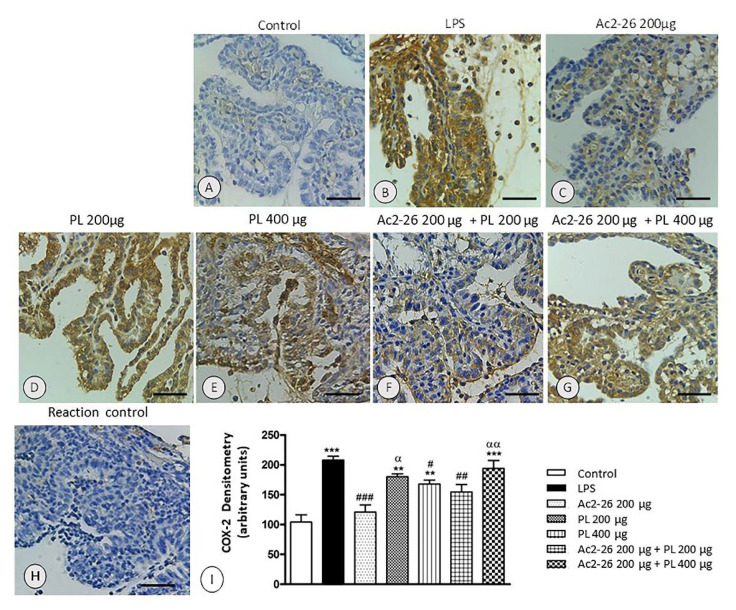
Expression of the COX-2 enzyme in ciliary processes in EIU. Absence of immunostaining in the control eyes (**A**). Strong immunostaining in the untreated, induced EIU animals (LPS) (**B**) and those treated with PL 200 μg (**D**) and Ac2-26 + PL 400 μg (**G**). Decreased expression after systemic treatments with Ac2-26 (**C**), PL 400 μg (**E**), and Ac2-26 + PL 200 μg (**F**). Absence of immunostaining in the control eyes (**H**). Counterstaining: hematoxylin, bars: 10 μm. Densitometric analysis of COX-2 (**I**). Results were obtained as mean ± SEM of the densitometric index of the eyes of control rats, untreated uveitis (LPS), and treated groups (Ac2-26 200 µg, PL 200 µg, PL 400 µg, Ac2-26 + PL 200 µg, and Ac2-26 + PL 400 µg) (*n* = 10 animals/group). *** *p* < 0.001 and ** *p* < 0.01 versus control; # *p* < 0.05, ## *p* < 0.01, and ### *p* < 0.001 versus LPS; αα *p* < 0.01 and α *p* < 0.05 versus Ac2-26 200 µg.

In the anterior ocular segment of the control rats, especially in iris, ciliary body, and ciliary processes (Figure 5A), the expression COX-2 enzyme was not detected. However, in the same regions, in the untreated EIU animals (Figure 5B) and after systemic treatments with PL 200 μg and Ac2-26 + PL 400 μg (Figure 5D,G), strong COX-2 immunolabeling was observed. The Ac2-26, PL 400 μg, and Ac2-26 + PL 200 μg groups showed reduced enzyme expression (Figure 5C,E,F), mainly in the singly peptide treated group. There was no immunoreactivity for COX-2 in the reaction control (Figure 5H), confirming antibody specificity.

Densitometric analyses corroborated the immunohistochemical observations (Figure 5I), reinforced the anti-inflammatory activities of PL in a dose-dependent manner, and showed that the combination of Ac2-26 and PL, especially at the higher dosage, inhibited the protective action of the peptide and PL singly administered.

### 3.4. Endogenous AnxA1 Increased during Inflammation in Ocular Tissues, but Ac2-26 Administered Singly or in Combination with PL at Lower Dosage Reduced AnxA1 Immunoreactivity

**Figure 6:**
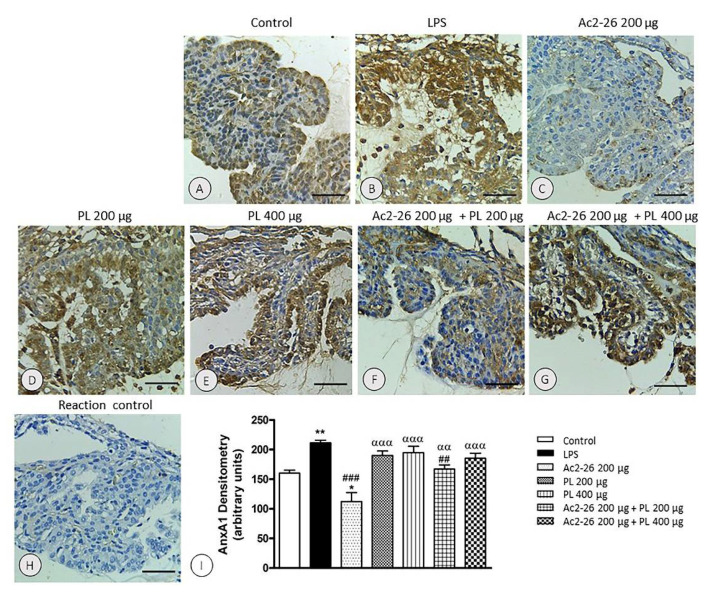
Expression of AnxA1 in ciliary processes in EIU. Increased expression after 24 h of induction of inflammation in the untreated (LPS) (**B**) and treated groups with PL (**D**,**E**) and Ac2-26 + PL 400 μg (**G**) relative to the control (**A**). Reduction of immunoreactivity after treatments with Ac2-26 (**C**) and Ac2-26 + PL 200 μg (**F**) compared to LPS. Absence of immunoreactivity in the control of the reaction (**H**). Counterstaining: hematoxylin, bars: 10 μm. Densitometric analysis of AnxA1 (**I**). Results were obtained as mean ± SEM of the densitometric index of the eyes of control rats, untreated uveitis (LPS), and treated groups (Ac2-26 200 µg, PL 200 µg, PL 400 µg, Ac2-26 + PL 200 µg, and Ac2-26+ PL 400 µg) (*n* = 10 animals/group). ** *p* < 0.01 and * *p* < 0.05 versus control; ## *p* < 0.01 and ### *p* < 0.001 versus LPS; ααα *p* < 0.001 and αα *p* < 0.01 versus Ac2-262 00 µg.

The immunohistochemical and densitometric analyses of AnxA1 expression in the anterior eye segment showed significant increase in the endogenous protein, especially in the ciliary processes, 24 h after uveitis induction in the untreated group (*p* < 0.001) compared to the control (Figure 6A,B,I).

AnxA1 immunolabeling remained increased after treatments with PL singly administered (Figure 6D,E,I) or in combination with Ac2-26 at 400 μg dosage (Figure 6G,I). In contrast, in the Ac2-26 (Figure 6C) and Ac2-26 + PL 200 μg groups (Figure 6F) decreased AnxA1 expression was observed relative to the LPS group (Figure 6I). In the group treated only with the peptide, the expression of the protein was also reduced compared to control group (*p* < 0.05). The specificity of the immunolabeling was confirmed by the reaction control (Figure 6H).

### 3.5. Expression of the fpr2 Receptor Is Modulated by Treatment with AnxA1 Peptide but Not with PL Singly Administered or Ac2-26 in Combination with PL 400 µg

**Figure 7:**
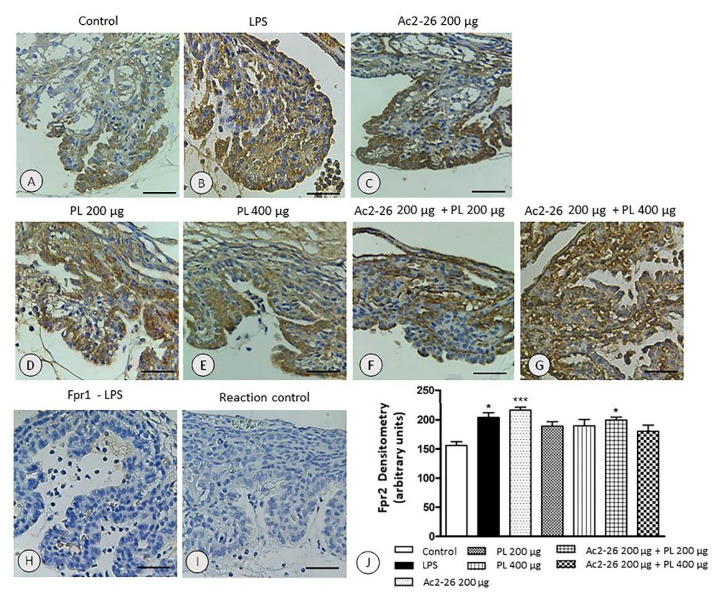
Specific expression of fpr2 in ocular tissues. Strong immunoreactivity for fpr2 in the LPS (**B**) and Ac2-26 (**C**) groups. Similar expressions were found among the control (**A**), PL (**D**,**E**), and Ac2-26 + PL (**F**,**G**) groups. Absence of labeling for fpr1 receptor (**H**) and in control of the reaction (**I**). Counterstaining: hematoxylin, bars: 10 μm. Densitometric analysis of fpr2 (**J**). Results were obtained as mean ± SEM of the densitometric index of the eyes of the control rats, untreated uveitis (LPS), and treated groups (Ac2-26 200 µg, PL 200 µg, PL 400 µg, Ac2-26 + PL 200 µg, and Ac2-26 + PL 400 µg) (*n* = 10 animals/group). *** *p* < 0.001 and * *p <* 0.05 versus control.

The expression of fpr2 receptor in ocular tissues overlapped the location of the AnxA1 with increased labeling in the LPS group (*p* < 0.05) (Figure 7B,J) and mainly in animals treated with AnxA1 peptide (*p* < 0.001) (Figure 7C,J) compared to the control (Figure 7A,J). However, after other treatments, there was no significant change in receptor expression (Figure 7D–G,J). In contrast, we observed no immunoreactivity for fpr1 (Figure 7H) in all the studied groups. The control of the reaction indicated antibody specificity (Figure 7I).

## 4. Discussion

Several investigations have explored the anti-inflammatory and protective activities of AnxA1 protein and its mimetic peptides, especially Ac2-26 [25,29]. In recent years, our group has researched the role of AnxA1 in the eye by means of in vivo and in vitro studies and highlighted the potential of the protein in controlling the ocular inflammatory process [16,17,19,34,35]. Recently, the understanding that AnxA1 could interact with PL [36] has opened up a new and exciting field of research. Thus, we proposed an investigation into the effects of PL administered alone in the EIU, and our findings indicate an important anti-inflammatory profile of PL in LPS-induced uveitis. Moreover, we evaluated the coadministration of Ac2-26 + PL in the experimental uveitis. We speculated whether their coadministration would have synergistic or antagonistic effects. It would be expected that the combination would enhance the action against uveitis, but the result proved to be the opposite. It follows from this result that both might also act in close proximity and that a possible peptide structure conformational change occurred.

Our initial analysis, as expected, showed that inflammatory stimuli induced by LPS released inflammatory mediators IL-1β, IL-6, TNF-α, and MCP-1 and increased expression of COX-2 by promoting disruption of the blood–ocular barrier and intense influx of leukocytes, reinforcing previous studies [17,31]. Neutrophils were the predominantly extravasated inflammatory cells, especially near ciliary processes. In EIU, neutrophil transmigration occurs at the base of the ciliary body, whereas the infiltrate of phagocytic mononuclear cells and lymphocytes occurs in the iris vessels [16,17]. The expression of *Toll-like receptor 4* (TLR4), preferably by cells in the anterior region of the eye, may explain the apparent susceptibility of anterior uvea to disruption of the blood–ocular barrier and development of uveitis [50,51].

Data obtained after systemic treatments with Ac2-26 and PL in EIU confirmed the anti-inflammatory action of AnxA1 mimetic peptide in experimental ocular inflammation [18,32], including uveitis, as previously reported by our research group [17]. However, as a novelty, our current results also indicated the protective effects of PL at 400 μg dosage and therefore a dose-dependent manner. Both Ac2-26 and PL at 400 μg dosage promoted decrease in the influx of neutrophils and monocytes into AqH and blood as well the reduction of the phagocytic mononuclear cells in the iris and ciliary body. Furthermore, the anti-inflammatory effects of the mimetic peptide and PL at higher dosage stimulated the reduction of IL-1β, IL-6, TNF-α, and MCP-1 levels, which are produced especially by neutrophils and phagocytic mononuclear cells [52,53].

Our findings in relation to PL in EIU are in agreement with other investigations that showed the anti-inflammatory potential of PL, such as in the reduction of ear edema induced by croton oil [54], analgesia and suppression of the stress response caused by pain in a dose-dependent manner [55], and decreased release of TNF-α and IL-1β in collagen-induced arthritis [56]. In particular, in LPS-induced-inflammation, PL suppressed leukocytes migration, TNF-α and IL-6 production, NF-κB and regulated extracellular kinases (ERK) 1 and 2 activation [46], and reduced mortality in sepsis [44]. Moreover, in LPS-induced neuroinflammation, PL protected dopaminergic neurons against inflammation by inhibiting microglial activation and decreasing levels of TNF-α, IL-1β, IL-6, and the production of ROS and NO [48] as well as inhibiting NF-κB and amyloidogenesis [49].

However, when Ac2-26 was administered in combination with PL, anti-inflammatory effects were abrogated, especially in combination with PL at 400 μg dosage. Interestingly, the groups that showed increased levels of the anti-inflammatory cytokine IL-10 were LPS and Ac2-26 + PL 400 μg, whereas treatment with peptide singly administered led to reduction of this cytokine level. In the model of allergic conjunctivitis, low levels of IL-10 were also observed after treatment with Ac2-26, and a significant increase in this cytokine occurred in AnxA1-null animals [32], indicating the importance of Th1/Th2 balance in the development of allergic inflammatory responses and suggesting that the protective role of AnxA1 in ocular allergy occurs through downregulation of both cytokine profiles. The same seems to happen in EIU.

The efficacy of Ac2-26 and PL at 400 μg dosage was also verified by the reduction in COX-2 proinflammatory enzyme expression. Again, the administration of PL at 200 μg dosage and the combination of Ac2-26 + PL 400 μg did not revert the inflammatory process. In a previous research, we had shown the exacerbated inflammatory response, characterized by COX-2 overexpression in the eyes of AnxA1-null mice, reinforcing the actions of AnxA1 in the resolution of ocular inflammation [17]. Concerning PL, the importance of its analogs in COX-2 inhibition after LPS induction was demonstrated in the RAW264.7 lineage of macrophages [38]. More recently, our research group showed that PL administration decreased expressions of COX-2, NF-κB, and neutrophil elastase and recovered lung tissues in a model of lung inflammation [42].

We studied the endogenous expression of AnxA1 in the different experimental groups, especially in the anterior eye segment (iris, ciliary body, and ciliary processes). The immunoreactivity for AnxA1 in the ocular tissues overlapped with the sites of TLR4 expression and production of inflammatory mediators [57,58]. Indeed, TLR4 in the eye is particularly expressed by epithelial cells (cornea and pigmented epithelia of the ciliary body), iris endothelial cells [50,51], and resident antigen presenting cells of the uvea [52,59]. Moreover, in uveitis, cytokines are produced mainly by inflammatory and endothelial cells as well as by corneal epithelial cells and retinal pigmented cells [57,58].

The immunohistochemical analysis of untreated EIU eyes showed an increase in the intensity of immunolabeling for AnxA1, corroborating our previous findings in ocular tissues [17,34] and ocular inflammatory cells [16]. The higher expression of AnxA1 was also observed in neutrophils in ocular toxoplasmosis in mice and culture of retinal pigmented cells infected with *Toxoplasma gondii* [30]. In contrast, in animals treated with Ac2-26 singly or in combination with PL at lower dosage, there was a decrease in immunoreactivity, probably due to a negative feedback mechanism, as hitherto observed in the uveitis [17] and allergic conjunctivitis [32] models.

The fact that endogenous AnxA1 is strongly induced by LPS has already been reported in other investigations and reinforces the action of AnxA1 as a proresolving mediator in inflammation [23,26,27]. In the systemic inflammatory reaction induced by LPS, higher AnxA1 expression was observed and associated with the combined actions of endogenous glucocorticoids IL-6 and TNF-α [60]. Intense increase in *AnxA1* gene activity and protein synthesis in hepatocytes, endothelial cells, and leukocytes at the beginning of the inflammatory process, followed by reduction in AnxA1 expression in the late phase of inflammation, was verified by means of the *LacZ* reporter gene in AnxA1-null mice after LPS endotoxemia [61]. Similar to our findings, AnxA1 expression increased during lung inflammation induced by LPS but decreased after peptide Ac2-26 treatment [62]. Moreover, in the model of LPS-induced pleurisy, glucocorticoid-induced leucine zipper (GILZ) deficiency was associated with an early increase in AnxA1, so the lack of endogenous GILZ during the resolution of inflammation was compensated by AnxA1 overexpression [63].

Although our results indicated that the combination of the peptide with PL promoted a decrease in the effects of Ac2-26, the dosage of 200 μg of PL still allowed the peptide actions in a moderate manner, which may explain the lower expression of the endogenous AnxA1 in this group. Modulation in the expression of AnxA1 after LPS inoculation found in this investigation reinforces the involvement of the protein in ocular tissue physiology during inflammatory [16,17], infectious [34], allergic [32,35], and autoimmune [18,20] processes in experimental models. Interestingly, the expression of AnxA1 remains increased after treatments with PL singly administered or at 400 μg dosage in combination with the peptide. This finding could reflect the results found by other researchers who used herbal medicines, as in rats induced with sepsis and treated with Xuebijing (XBJ) [64] and culture of lung tumor cells administered with *Camellia simensis* [65]. Similar to our results, in a model of lung inflammation induced by cigarette smoke, PL administration promoted increased expression of AnxA1, concomitant with the reduction in COX-2 [42].

Following the study, we investigated the expression of fpr1 and fpr2 receptors in all groups. In previous studies on inflamed ocular tissues, it was shown that the expression of fpr2 perfectly overlapped the distribution of AnxA1 and that it was increased after treatment with Ac2-26 [17,34]. Again, our results strengthen the possible specificity of the AnxA1/fpr2 interaction as the expression of the fpr1 receptor did not occur in any of the experimental groups. In contrast, intense expression of both fprs were detected in conjunctival epithelial cells in an allergic conjunctivitis model [35], but the lack of AnxA1 protein in the ovalbumin-sensitized mice produced a marked increase only in fpr2 expression.

In addition, we found that the fpr2 expression was not altered by PL singly administered or in combination with Ac2-26, suggesting that the attenuation of the protective effects of mimetic peptide by interaction with PL probably is not related to receptor expression changes. In the light of these data and our previous findings about the interaction between Ac2-26 and PL [36], we speculate that conformational alteration may have occurred in the Ac2-26 + PL complex and may have interfered with the fpr2 binding receptor, which impaired the anti-inflammatory actions of the peptide. This reasoning is supported by recently reported results as we demonstrated that the interaction of PL with the AnxA1-derived peptide Ac2-26 occurred spontaneously, was enthalpically driven, and that the forces governing the interaction were hydrophobic. Moreover, Ac2-26 peptide binds to PL via two hydrogen-bonding interactions at lysine 9 but not at tryptophan 12 [36]. Previous data from our group have allowed us to report that the anti-inflammatory activities of AnxA1 occur after a specific serine phosphorylation event in the N-terminal region [17]. Therefore, as the interaction between Ac2-26 and PL occurs on tyrosine, the serine site, which is an important post-translational modification related to the translocation of AnxA1 from cytoplasm to cell surface [28], remains free for phosphorylation.

**Figure 8:**
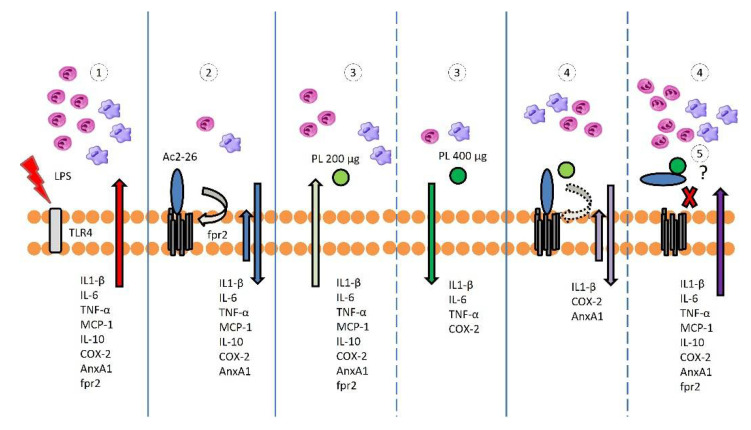
Schematic model of Ac2-26 and PL actions in EIU. (**1**) LPS triggers the release of cytokines (IL1-β, IL-6, IL-10, TNF-α, MCP-1), leukocyte influx (neutrophils and monocytes), and the production of endogenous COX-2, AnxA1, and fpr2. (**2**) These inflammatory responses are mitigated by Ac2-26, mediated by fpr2 receptor, and (**3**) PL, especially at a higher dosage. (**4**) The coadministration of Ac2-26 and PL abrogates the anti-inflammatory effects of the singly administered compounds. (**5**) Conformational changes on the Ac2-26 peptide due to its interaction with PL may impair its binding to the fpr2 receptor. Larger arrows directed upwards or downwards indicate an increase or decrease, respectively, of cytokines and endogenous proteins produced by the cell. Smaller arrows pointing upwards indicate increased expression of fpr2. Outside the cell, pink shape represents neutrophils, purple shape represents monocytes, light green circle depicts PL at 200 µg dosage, dark green circle depicts PL at 400 µg dosage, and blue oval represents Ac2-26.

At first, this could indicate that PL does not affect the action of endogenous AnxA1. This thought was supported by our current findings, which showed increased expression of AnxA1 when PL was administered alone. However, with coadministration in vivo, Ac2-26 and PL promoted the reversal of the anti-inflammatory effects when administrated alone (Figure 8). Moreover, the higher the concentration of PL, the greater the possibility of interaction of this molecule with Ac2-26 and, consequently, the lower the anti-inflammatory response. These findings indicate that the impairment of the anti-inflammatory action of Ac2-26 may be related to a conformational change, which prevents the peptide from binding to the fpr2 receptor. Relevantly, the PL sequestered by the complex is also prevented from entering the cells and performing its anti-inflammatory role. Thus, there is a competition between Ac2-26 and PL in the anti-inflammatory action against uveitis. This important result implies there are other factors to be considered, such as molecular weight, size of the molecules, charge distribution on the peptide, possible conformational structure adopted by the peptide during the action, and localization of the interaction site. Thus, a reasonable next step will be studies requiring new approaches, both from the point of view of action against uveitis as well as experimental evidence.

## 5. Conclusions

Our study sheds light on the protective effects of PL, revealing it as a potential therapeutic target in ocular inflammation. Furthermore, the results show that Ac2-26 + PL combination abrogates the anti-inflammatory actions of Ac2-26 and PL singly administered.

## Data Availability

Data available on request due to restrictions.
